# Characterization of the microscopic tribological properties of sandfish (*Scincus scincus*) scales by atomic force microscopy

**DOI:** 10.3762/bjnano.9.243

**Published:** 2018-10-02

**Authors:** Weibin Wu, Christian Lutz, Simon Mersch, Richard Thelen, Christian Greiner, Guillaume Gomard, Hendrik Hölscher

**Affiliations:** 1 Institute of Microstructure Technology (IMT), Karlsruhe Institute of Technology (KIT), H.-v.-Helmholtz Platz 1, 76344 Eggenstein-Leopoldshafen, Germany; 2 Institute for Applied Materials (IAM), Karlsruhe Institute of Technology (KIT), Strasse am Forum 5, 76131 Karlsruhe, Germany; 3 Light Technology Institute (LTI), Karlsruhe Institute of Technology (KIT), Engesserstrasse 13, 76131 Karlsruhe, Germany

**Keywords:** biotribology, frictional properties of reptile scales, sandfish, *Scincus scincus*

## Abstract

Lizards of the genus *Scincus* are widely known under the common name sandfish due to their ability to swim in loose, aeolian sand. Some studies report that this fascinating property of sandfish is accompanied by unique tribological properties of their skin such as ultra-low adhesion, friction and wear. The majority of these reports, however, is based on experiments conducted with a non-standard granular tribometer. Here, we characterise microscopic adhesion, friction and wear of single sandfish scales by atomic force microscopy. The analysis of frictional properties with different types of probes (sharp silicon tips, spherical glass tips and sand debris) demonstrates that the tribological properties of sandfish scales on the microscale are not exceptional if compared to snake scales or technical surfaces such as aluminium, Teflon, or highly oriented pyrolytic graphite.

## Introduction

**Figure 1:**
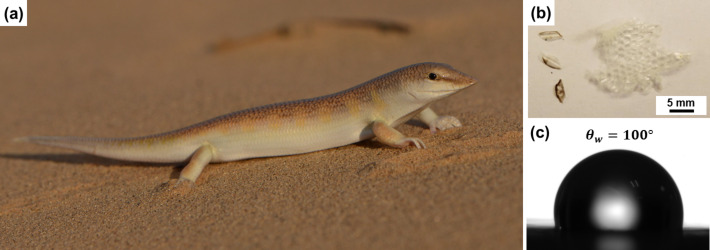
(a) Photograph of a sandfish (*S. scincus*) in its natural habitat (copyright Gerrit Jan Verspui). (b) Photograph of scales from moulted sandfish skin (*S. scincus*) examined in this study. Cut parts of the moulted skin or singled scales were used for all measurements. (c) The typical contact angle of a single sandfish scale is about 100° (droplet volume 1 µL).

Areas with loose, aeolian sand in the deserts of North Africa and the Arabian Peninsula are the habitat of the lizard *Scincus scincus* [1] (see Figure 1a). It hides from predators by burying in sand within seconds. This defence strategy is also known from other reptiles [1]. *S. scincus,* however, is not only able to bury, it can also “swim” and travel reasonable distances in sand [2–4]. Velocities of up to 30 cm/s and distances of several meters are reported [3]. This fascinating feature is the origin of the common name sandfish for this lizard being adapted to its environment [5]. Studies analysing the locomotion of sandfish in granular media via nuclear magnetic resonance (NMR) imaging [2] or high-speed X-ray imaging [4] indeed show that the movement of a sandfish resembles that of swimming fishes.

It is surprising that sandfishes manage to bury and swim in sand without visible wear on their skin [2–3,6–9]. This contradicts everyday experience because a tiny grain of sand easily scratches practically any technical surface even hard ones such as glass or steel. The widely applied sandblasting, for example, is based on this effect. The sandfish, however, moults its skin only every two to three months [6], and we are not aware of any report of observable wear on sandfish skin caused by its swimming in loose sand. Rechenberg [3,7–8] and Baumgartner et al. [6,9,12] conducted pioneering studies analysing friction and wear of sandfish skin applying a granular friction approach introduced by Rechenberg [7]. Sand is poured through a funnel directly on the tilted body of the animal or surface under observation. The angle at which the sand stops to slide off the animal or surface is the granular friction angle. This granular tribometer is of high practical value for field studies where classical tribometer experiments with animals are challenging. Rechenberg’s studies [3,7–8] and subsequent studies of Staudt et al. [9,11] revealed that this granular friction angle of preserved sandfish is about θ = 21° (corresponding friction coefficient μ_gr_ = tan θ = 0.38). This value is indeed lower than those of other technical surfaces such as aluminium (θ = 25°), steel (θ = 26°) or Teflon (θ = 35°), which were examined with the same sand and setup [3]. Interestingly, granular friction angles reported for closely related but not sand-swimming lizards such as the banded skink (*Scincopus fasciatus*, θ = 31°) or the Berber skink (*Eumeces schneideri*, θ = 35°) are considerably higher than that of the sandfish [9,12]. Nonetheless, it has to be taken into account that these granular friction angles were determined with loose granular sand where no external load is applied. Sharpe et al. [13] sedated animals, put them on a tilted flat covered with a monolayer of granular particles, and determined the angle at which the animal started to slide in forward direction on its ventral scales. The static friction coefficient µ_st_, determined in this more classical way, was higher for sandfish (*S. scincus*, µ_st_ = 0.19) than for the shovel-nosed snake (*Chionactis occipitalis*, µ_st_ = 0.11) which also does sand swimming.

Rechenberg [3] observed comb-like nanostructures on the sandfish as well as on the Kenyan sand boa (*Eryx colubrinus*) and the wedge-snouted skink (*Sphenops sepsoides*). Therefore, he assumed that these are the origin of the favourable frictional properties of reptiles living in a sandy environment. Klein et al. [14] speculated that a material gradient in the snake integument minimizes damage during locomotion. However, as pointed out by Baumgartner et al. [6,10] the comb-like nanostructure of the sandfish is found only on dorsal scales and is missing on ventral scales. Moreover, both types feature a similar friction coefficient. Finally, such a comb-like structure can be found on many reptiles even on those that do not sand-swim or live in a different environment [15]. Consequently, it is highly unlikely that the surface structure of the scales is responsible for the observed low abrasion.

Baumgartner and co-workers [10–11,16] measured adhesion by atomic force microscopy (AFM) on scales of *S. scincus* and observed extremely low values. They analysed the chemical composition of the scales and concluded that the low adhesion, and the resulting low friction and high abrasion resistance, is a material property caused by glycosylated β-keratins in the scales. Neutral glycans with five to nine mannose residues in sandfish scales are supposed to act as low-density spacers separating sand particles from the dense scales thereby reducing van der Waals forces [16]. Even a glycosylated technical surface showed a reduced granular friction coefficient [16].

Here, we analyse the tribological properties of single scales of sandfish (*S. scincus*) by atomic force microscopy and microtribometer experiments. Using different types of AFM probes we do not observe favourable frictional properties of sandfish scales if compared to technical surfaces with tribological relevance. Even a direct comparison with scales of various snakes does not reveal superior features. Experiments with a microtribometer, where the same types of samples were paired against a 1 mm diameter sapphire ball, confirm this observation on a much larger scale as probed by AFM. Neither adhesion nor friction coefficient of sandfish scales are found to be lower than other surfaces if measured with an AFM. Also, the wear resistance recorded with an AFM tip is not outstanding. Microtribometer experiments do neither reveal exceptional frictional properties. We, therefore, conclude that the fascinating ability to swim in sand without observable abrasion is not solely caused by the scales of sandfish. Other, at least additional, mechanisms are likely to be involved.

## Experimental

Moulted sandfish skin collected from kept animals was cut in small pieces or scales were singled before sample preparation (Figure 1b). In some cases it is possible to distinguish between dorsal and ventral scales through their different colour and microstructure. Pieces of skin from the dorsal side have some darker areas while the ventral side is completely opaque. Furthermore, the dorsal scales feature comb-like microsteps while the ventral scales a nearly planar as described by Baumgartner and co-workers [10]. All results presented here were measured with scales from *S. scincus* (provided by G. Gassner, Natural History Museum, Vienna, Austria). These were not tested for their content of glycans [16]. For comparison, we also analysed technical materials such as graphite, Teflon, poly(methyl methacrylate) (PMMA), polyether ether ketone (PEEK), aluminium and silicon. In addition to that, we examined scales from four different snakes, which were also collected after skin-shedding (provided by G. Gomard, KIT). *Spalerosophis diadema cliffordii* (Clifford's diadem snake) is a psammophile snake living in a sandy environment but not in sand dunes like sandfish. *Echis pyramidum* (Egyptian saw-scaled viper) lives near sandy environments while *Pantherophis guttatus* (Eastern corn snake) and *Naja atra* (Chinese cobra) live in various habitats and they are not particularly psammophile. All samples of scales were stored and measured in an environment with controlled temperature (21–23 °C) and humidity (50–70%).

All AFM experiments were conducted with a Dimension Icon AFM (Veeco Inc., USA). The topography of the samples was measured in tapping mode while adhesion force, friction, and wear analysis were conducted in contact mode. No extra treatment was applied to the scales before imaging. Spring constant and deflection sensitivity of all cantilevers (All-in-One-Al, BudgetSensors) were determined with the thermal tune method integrated into the corresponding AFM software. Normal load and lateral force were calibrated according to the procedure described by Schwarz and co-workers [17]. The ramp rate of the adhesion measurements was set to 1 µm/s. Microscopic friction was measured by scanning sample surfaces with a scan size of 20 µm × 20 µm and a defined loading force *F*_load_ while recording the lateral forces acting on the tip apex. Averaging these values gives the averaged frictional force <*F*_fric_>. The corresponding frictional coefficient μ was obtained by subsequently fitting the data with *F*_fric_ = *F*_ad_ + µ·*F*_load_.

Cantilevers and the cross section of a sandfish dorsal scale were imaged by scanning electron microscopy (SEM, SUPRA 60 VP, Zeiss, Germany). Sandfish scales were sputtered with 20 nm of silver before imaging while the probes were not sputtered in order to prevent unwanted changes of the surface properties for the adhesion measurements. Therefore, a low working distance between probes and SEM detector and a low acceleration voltage between 1 and 1.5 kV were used to enable the SEM investigation.

**Figure 2:**
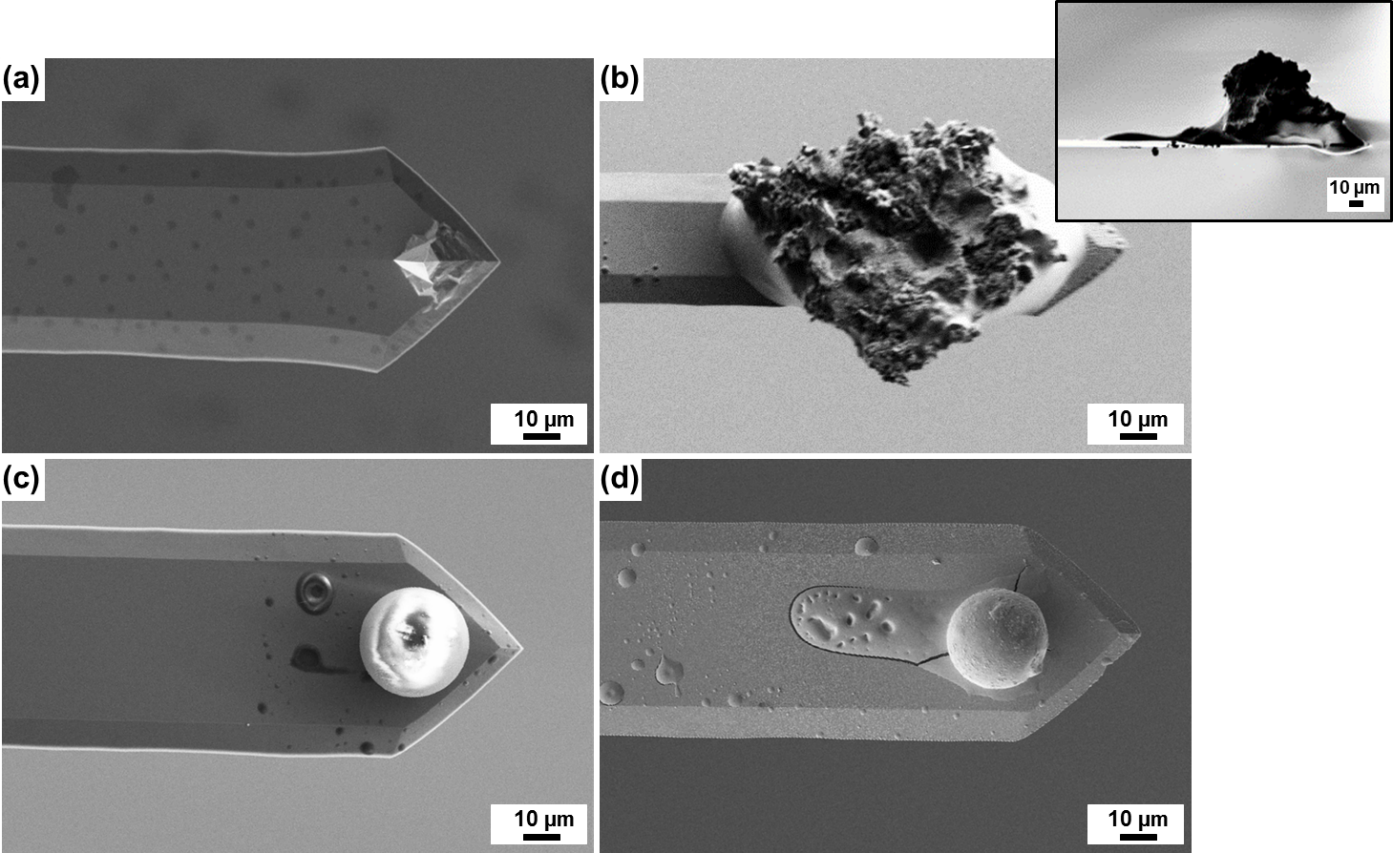
SEM images of some probes used in this study. (a) Sharp tip of a conventional AFM cantilever made from silicon. (b) Sand particle glued to the end of a tipless cantilever (“sand probe”). The inset is a side view. (c) Glass sphere glued to the cantilever end (“spherical probe”). (d) Spherical glass probe coated with copper (“spherical probe with Cu coating”).

In addition to conventional sharp silicon tips (Figure 2a), we prepared various types of probes for the adhesion measurements. For that, we glued tiny sand debris as well as glass spheres with diameters of 20 or 40 µm to the end of tipless cantilevers (All-in-One-TL, BudgetSensors) using the procedure described by Mak and co-workers [18]. Depending on the glued probe we call them “sand probe” (Figure 2b) or “spherical probe” (Figure 2c) in the following. Some spherical probes were sputter-coated with a 50 nm thick metal layer of copper or tungsten to obtain spherical probes with different surface energy (Figure 2d).

The microtribometer experiments were performed with our custom-built reciprocating linear setup similar to the one described elsewhere [19]. The different materials tested were paired against polished sapphire spheres with a diameter of 1 mm provided by Saphirwerk AG (Bruegg, Switzerland). The normal load for all experiments was 0.1 N and the sliding speed was 0.5 mm/s. The number of reciprocating cycles was ten. Friction force was measured with a strain gauge-based system and recorded with a custom-programmed LabView (National Instruments, Austin, USA) code. The tests were conducted at room temperature and in air with 50% relative humidity. Sample preparation for the non-biological samples relied on grinding with SiC papers of #800 down to #4000 grid. Mechanical polishing was carried out with a 3 µm diamond suspension for 5 min and with a 1 µm diamond suspension for 8 min (DP-suspension M products purchased from Struers, Willich, Germany). This procedure resulted in scratch-free surfaces and a surface roughness of *R*_a_ < 0.01 µm, determined by optical profilometry (Sensofar Plµ neox, Barcelona, Spain).

The water contact angle of sandfish scales was measured with the sessile drop method using an OCA 40 system with the corresponding SCA20 software (DataPhysics Instruments, Germany)

## Results and Discussion

It is well-known that many parameters influence the frictional properties of surfaces. Comparable small variations in structure or chemistry may lead to drastic changes in friction or wear [20]. We, therefore, analyse the topography, adhesion, frictional coefficient, and wear resistance of sandfish scale by atomic force microscopy applying several types of probe shape and material. In order to allow for a meaningful comparison we determined the tribological parameters of snake scales and technical surfaces with the same probes, too.

### Structural properties of sandfish scales

**Figure 3:**
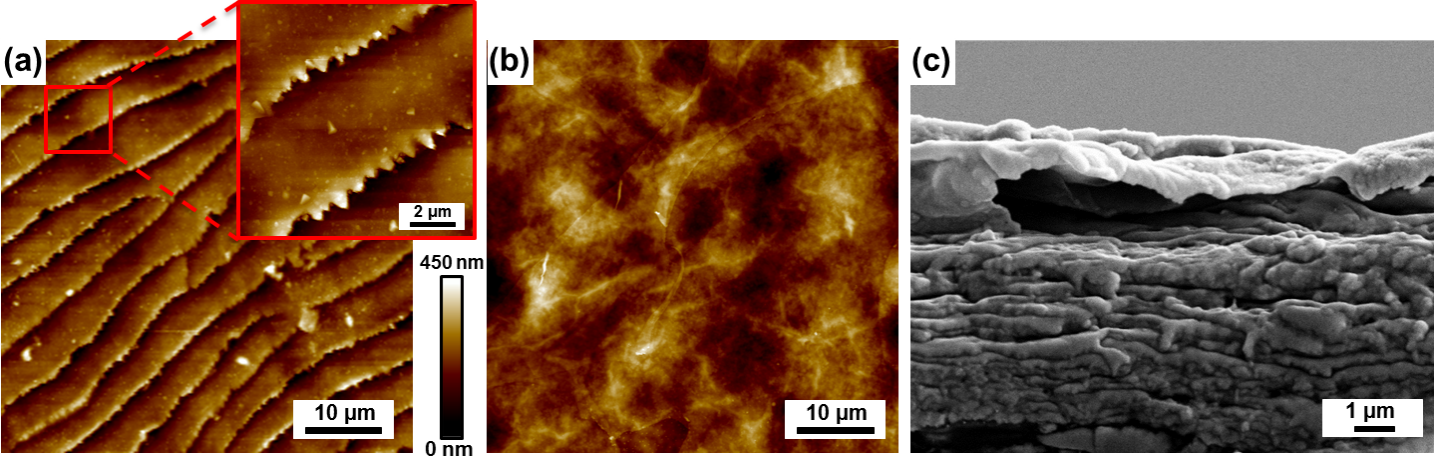
Structure of the analysed *S. scincus* scales. (a) The topography of a dorsal scale measured by atomic force microscopy reveals a structure of steps, which have a saw-tooth like shape magnified in the inset. (b) The topography of a ventral scale does not reveal steps. However, tiny scratches are sometimes visible. (c) A cross section of a dorsal scale recorded by scanning electron microscopy suggests that sandfish scales have a layered internal structure.

Figure 3a,b shows the topography of sandfish dorsal and ventral scales recorded by atomic force microscopy. On the dorsal scale a structure of steps with comb-like structures is observed in accordance with previous reports [6,10]. The average distance between two neighbouring steps is approximately 5 µm while the height of the steps is about 250 nm. The ventral scales, however, feature no recognizable steps and are comparably smooth. Nonetheless, larger images frequently reveal very fine groves, which might originate from scratches. A cross section of a dorsal scale imaged by electron microscopy is displayed in Figure 3c and shows an inner structure that suggest that the scale consists of several thin layers.

### Wetting properties

Some studies [10–11] report very low or nearly vanishing adhesion on scales of *S. scincus*. Low adhesion is a sign of low surface energy, which typically coincides with high contact angles [21]. As shown in Figure 1c, however, we observe contact angles of about 100° on single sandfish scales with small water droplets of 1 µL. Using larger volumes of 5 µL the water droplet gets in contact with several scales and the tissue between neighbouring scales. In this case, initial contact angles are smaller (92°) and decrease with time to values of about 80° to 60° after 10 min. We conclude that in the latter case the water spreads between the scales into the tissue connecting the scales. This observation coincides with other studies showing the same trend for sandfish and other reptiles, which optimized this mechanism to harvest water in their extremely dry environment [22]. Nonetheless, these contact angles are not unusually high compared to other reptiles or insects and do not suggest low surface energy or low adhesion. We, therefore, examined the adhesion of sandfish scales in more detail.

### Adhesion properties

**Figure 4:**
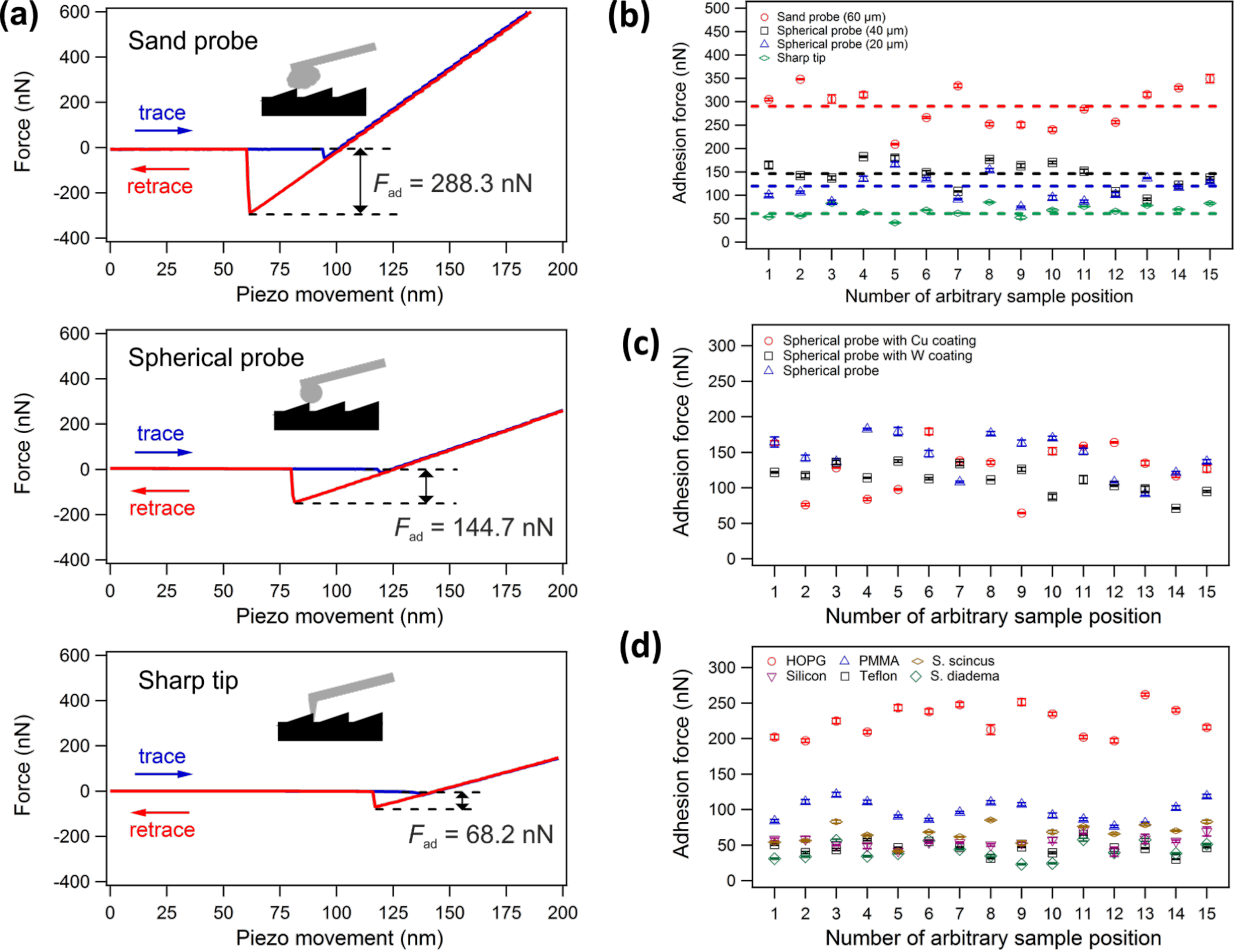
(a) Typical force–distance curves obtained with sand probe, spherical glass probe and sharp silicon tip, respectively. (b) Adhesion force obtained with four probes at 15 arbitrarily chosen positions on a sandfish scale. The adhesion force was measured ten times at each position and the error bars correspond to the statistical error. The dashed lines represent the overall average adhesion of all 150 measurements obtained with each probe, respectively. (c) The same experiment with spherical glass probes with a diameter of 20 µm without or with Cu or W coating reveals no significant dependence of adhesion on the coating. (d) Adhesion forces measured on different types of samples with a sharp tip reveal that the adhesion of the analysed sandfish scales is not significantly lower as that of other materials such as Clifford’s diadem snake (*S. diadema*) or technical surfaces such as Teflon.

Several different types of AFM probes were utilised to measure the adhesion force on dorsal scales. Figure 4a reviews three arbitrarily chosen force-vs-distance curves obtained with a sand probe, spherical probe and sharp tip. All curves feature a typical shape [23]. During the approach of the cantilever towards the sample (trace) the tip–sample force is almost zero and shows a small negative peak when tip and sample come into contact. After that the force increases linearly. During retraction the force decreases in a linear way before the tip is pulled off. This distinguished negative peak corresponds to the adhesion force *F*_ad_ marked in all graphs in Figure 4a. The adhesion peak for the sharp silicon tip is smallest (68.2 nN) but clearly visible. As it can be expected the adhesion peak increases with the contact area, and the spherical probe with a diameter of 40 µm has significantly larger adhesion force (144.7 nN) while the sand probe with an approximate diameter of 60 µm has the largest value (288.3 nN).

Figure 4b summarizes the adhesion analysis obtained with four different types of probes measured at 15 arbitrarily chosen positions. Ten force-vs-distance curves were recorded at each position (different for every probe). The error bars correspond to the statistical error. The dashed lines represent the respective average adhesion which increases with probe size: 67.3 nN (sharp tip), 121.0 nN (spherical probe 20 µm), 145.4 nN (spherical probe 40 µm), 290.8 nN (sand probe). Applying the same experimental procedure we measured the adhesion between a sandfish scale and two spherical probes with a diameter of 20 µm coated with copper (105.0 nN) and tungsten (115.5 nN). Figure 4c summarizes the results indicating that the metal coating influences the adhesion values only moderately compared to probe size, i.e., contact area.

In order to compare these values to other materials we conducted additional adhesion experiments with a sharp silicon tip on scales of *S. diadema* and on surfaces of some tribological relevance (PMMA, Teflon, highly oriented pyrolytic graphite (HOPG) and silicon). Figure 4d provides the averaged adhesion forces (*n* = 10) on 15 arbitrarily chosen positions recorded on every sample mentioned above. These measurements reveal that the averaged adhesion forces on sandfish *S. scincus* (67.3 nN) are a little larger than on silicon (54.2 nN), Teflon (46.1 nN) or *S. diadema* (41.3 nN) while adhesion on PMMA (98.2 nN) is considerably higher. Interestingly, the adhesion on HOPG (225.2 nN) is much higher, nearly 3.4-times of that of the sandfish scale. Nonetheless, the adhesion forces on sandfish scales are not found to be exceptionally low.

**Figure 5:**
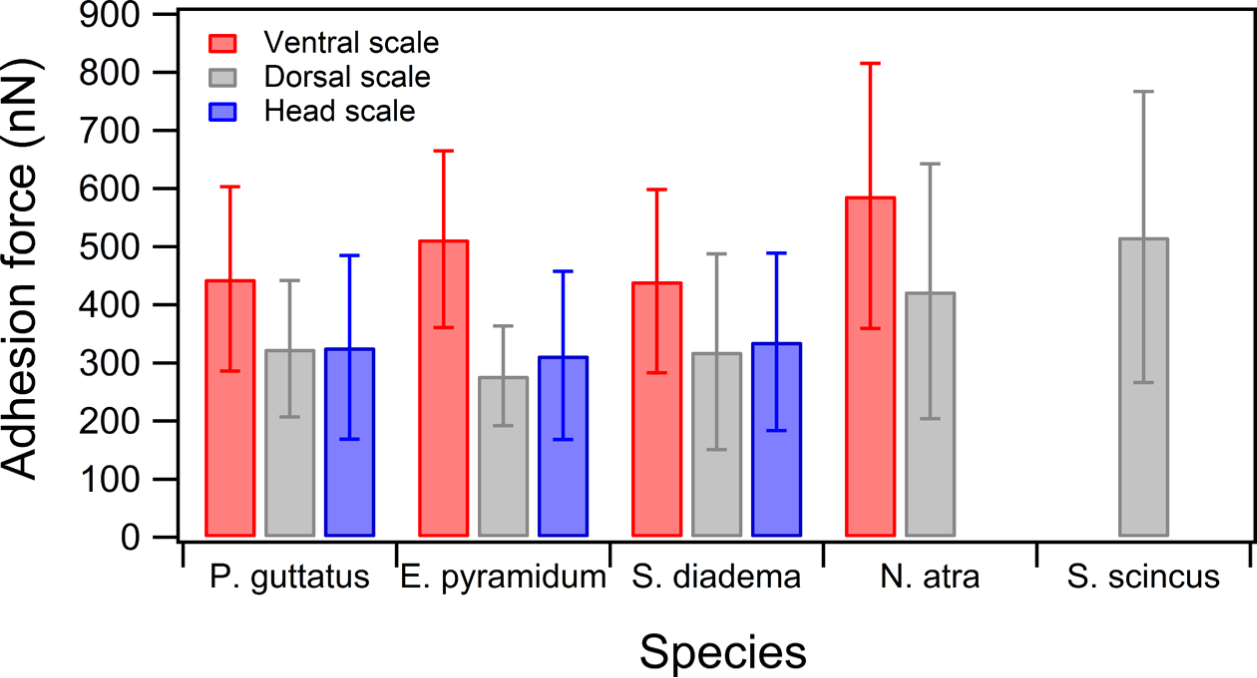
Direct comparison of the adhesion force measured with a sand probe on the scales of four species (*P. guttatus, E. pyramidum, S. diadema,* and *N. atra*) and dorsal scales of sandfish (*S. scincus*). Each bar corresponds to five force-vs-distance curves on fourteen different positions, i.e., *n* = 70 measurements.

We extended our analysis by measuring adhesion with a sand probe also on scales of four snakes (Figure 5). The scale samples were taken from the ventral, dorsal, and head area of *S. diadema cliffordii*, *E. pyramidum*, *P. guttatus*, and *N. atra*. For these snakes, adhesion forces on dorsal scales are smaller than on ventral ones but nearly equal to that on the head. Although *S. diadema* and *E. pyramidum* are snakes living in or near sandy environments we observe no difference to *P. guttatus* and *N. atra,* which are not psammophile. The adhesion force on scales of sandfish, however, is interestingly larger than that of all other examined snakes. This outcome demonstrates again that the adhesion of the analysed sandfish scales from *S. scincus* is not exceptional low as it might be assumed to explain low granular friction during sand swimming.

### Friction properties

Frictional properties of technical materials are generally described by the macroscopic frictional coefficient µ, which is the ratio between friction and applied load (µ = *F*_fric_/*F*_load_) of two bodies in contact. For a sandfish swimming in sand, however, there are numerous microscale contacts inducing friction without a defined load. The friction angle measurement introduced by Rechenberg [7] provides a simplified method to estimate a granular frictional coefficient (µ_gr_ = tan θ) with sand but it does not allow for a classical load-vs-friction analysis. We, therefore, conducted microscopic measurements recording friction-vs-load for various probes and surfaces. Fitting a straight line to the data we obtained the friction coefficient from the gradient of this fit.

**Figure 6:**
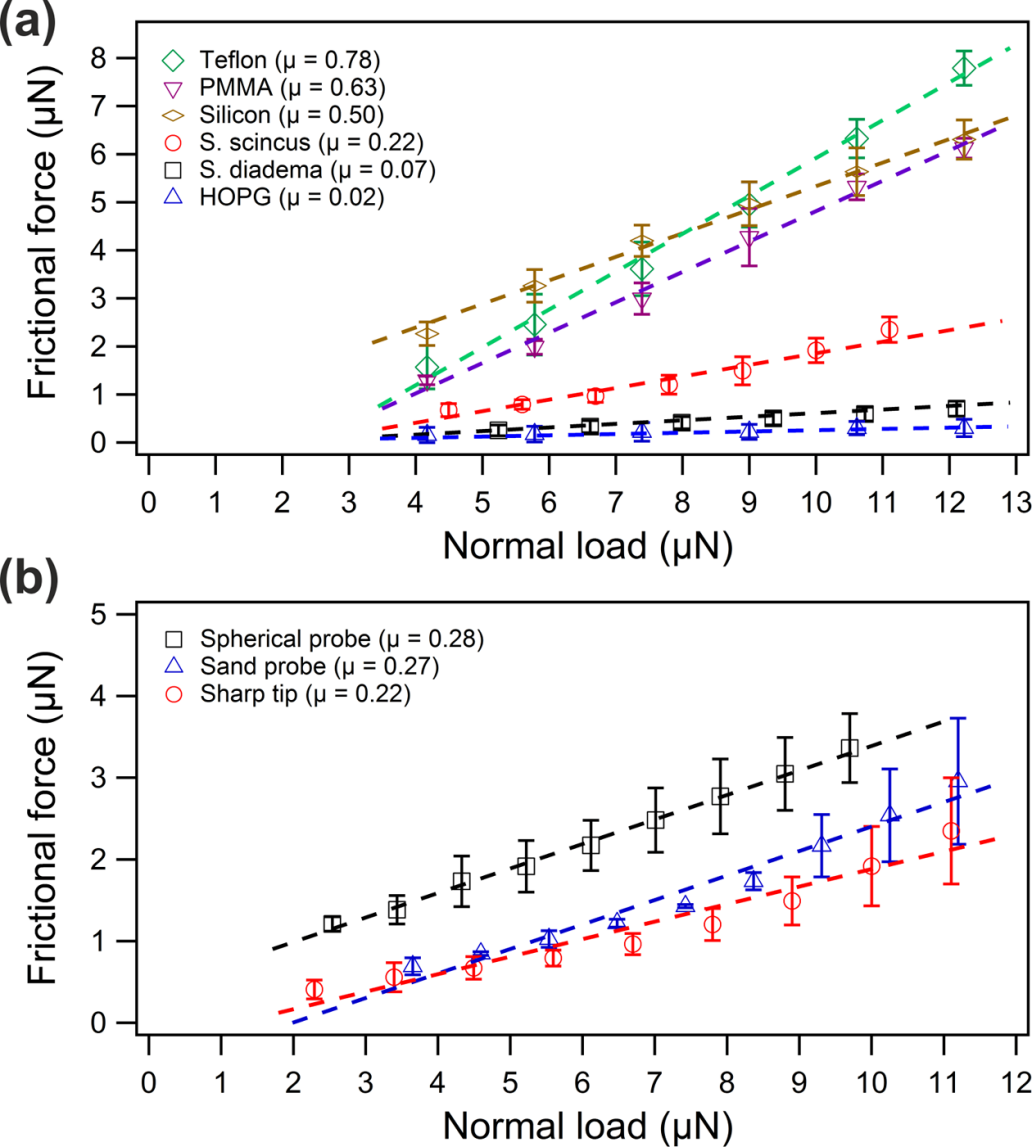
(a) Frictional force as a function of the normal load measured with a sharp silicon tip on a dorsal sandfish scale and five other sample surfaces. All data was obtained with the same sharp silicon tip. (b) Frictional force as a function of the normal load measured on a sandfish scale with a sharp silicon tip, a sand probe and a spherical glass probe. The dashed lines in the plots represent linear fits to the respective data sets of each material. The resulting gradient represents the microscopic frictional coefficient µ given in the legends.

Figure 6a displays the friction-vs-load curves of the investigated samples comprising Teflon, PMMA, silicon, sandfish, *S. diadema* and HOPG. All of these measurements were conducted with the same sharp silicon tip. For each sample, we measured a friction loop [17] for each load value and calculated the corresponding averaged friction. In this way, we average between forward and backward friction and neglect the anisotropy of friction due to the comb-like structure [24]. Three different positons on each sample were recorded and the averaged frictional force (data points in Figure 6) and the corresponding standard deviations (error bars in Figure 6) were calculated. Comparing the data for the technical surfaces it is evident that friction on Teflon, PMMA and silicon is larger than on HOPG. Since HOPG is a well-known dry lubricant this outcome can be expected. Interestingly, friction on *S. diadema* is nearly as small as on HOPG. Friction on sandfish scales, however, is found to be between these two groups. The dashed lines in Figure 6 correspond to the above-mentioned linear fit and the resulting frictional coefficients μ_AFM_ are provided in the legends. Among these samples, the largest and smallest frictional coefficients are observed on Teflon (0.78) and HOPG (0.02), respectively. The values for PMMA (0.63) and silicon (0.50) are larger than that for sandfish (0.22) and *S. diadema* (0.07). The microscopic frictional coefficient for sandfish scales measured with a sharp silicon tip is significantly lower than that for technical materials such as Teflon, PMMA and silicon but considerably higher than that for HOPG and a psammophile snake like *S. diadema*. This outcome shows that sandfish scales exhibit good but no excellent frictional behaviour at the microscale.

As this outcome is different to the results obtained with the granular-friction method [3,7–9] we wondered how friction might be influenced by the applied tip. We, therefore, utilised sand debris and a glass sphere as tip providing larger contact areas. The resulting friction-vs-load curves recorded with these two probes are displayed in Figure 6b. As for Figure 6a, we measured at three different spots for each normal load value. Fitting the frictional coefficients as before, we obtained µ values for sand debris (0.27) and spherical glass probe (0.28), which are slightly larger than those for the sharp silicon tip (plotted again in Figure 6b for comparison). Consequently, we can conclude that microscopic friction on sandfish scales is low but not exceptionally low as it might be expected.

### Scratch-resistance properties

Sandfish swim in sand grains and these particles close to the epidermis may act as a third abrasive when caught between the body and the rest of the sand, leading to a classical three-body abrasion system. Previous studies based on the granular approach indicated that sandfish skin got less damage and resisted wear much better than Teflon, glass or even steel [3,7–8]. We, therefore, investigated the abrasion resistance of dorsal sandfish scales on the microscale and compared it with other surfaces.

Scratch resistance experiments were conducted on various samples including sandfish scales, *S. diadema* scales, PMMA, Teflon and aluminium. In order to provoke some wear, we increased normal load with the aim to scratch the surface of the samples. To achieve such a large normal load, we utilized cantilevers with a nominal spring constants of 40 N/m. To avoid that tip wear influences the scratching tests, we started every experiment with a fresh cantilever with a pristine tip. On each sample, we scratched nine small areas with the same size (5 µm × 5 µm), number of scan lines (128 × 128) and scan velocity (0.8 Hz) but systematically increased the load for each subsequently scanned area.

**Figure 7:**
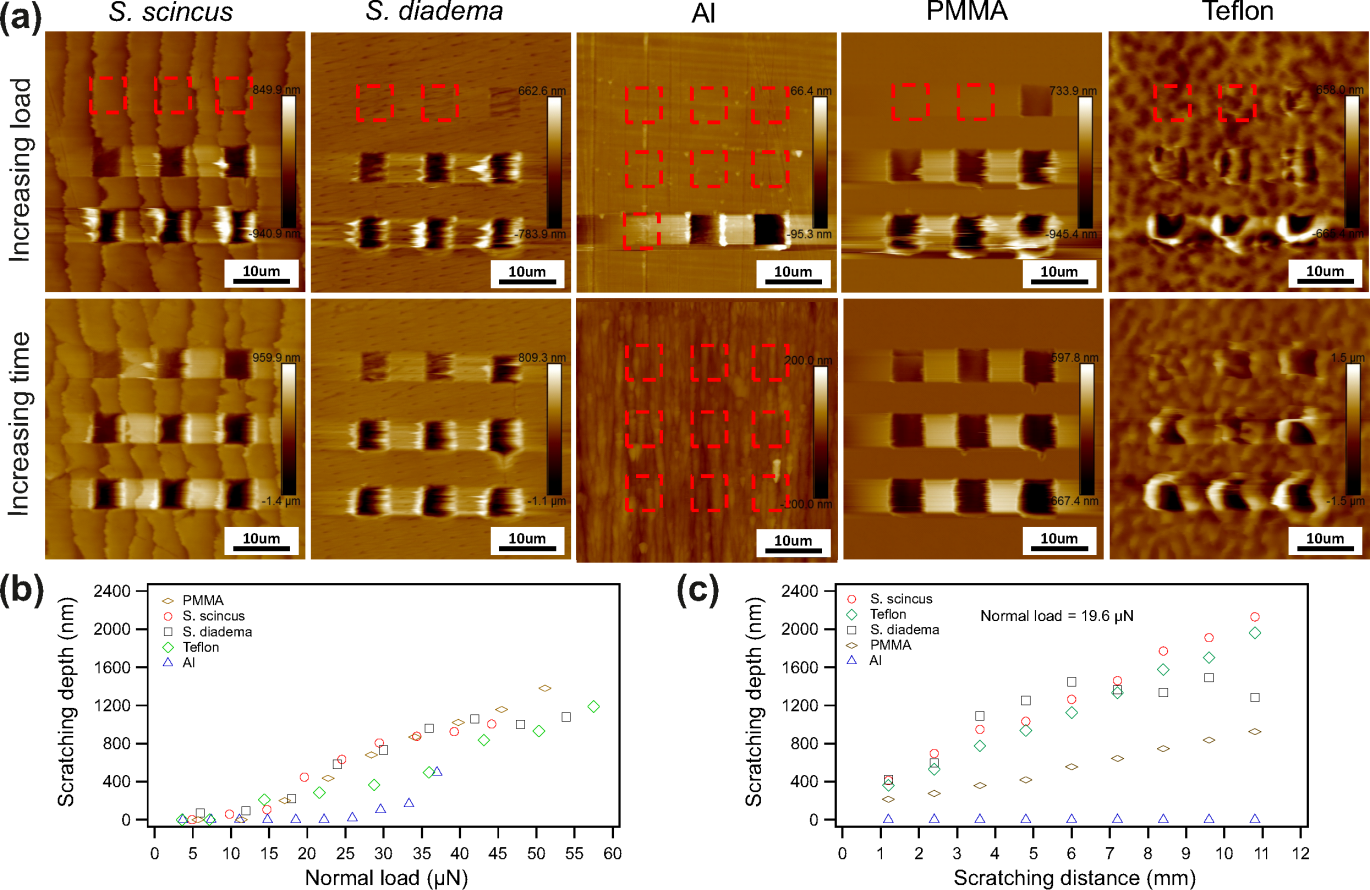
(a) Wear experiments recorded on five different materials with hard cantilevers (spring constant of approx. 40 N/m) and sharp silicon tips. Nine areas (5 µm × 5 µm) were scratched on each sample with increasing load or fixed load and increasing time. Every experiment was started with a pristine sharp tip cantilever. The red rectangles mark areas where no wear was observed. The top line shows the wear pattern with increasing normal load in steps (left to right and top to bottom). In this way the loading force increased to 35–60 µN in the lower right corner while the other scratching conditions were fixed. For the scratch test in the bottom line the normal load was fixed to 19.6 µN but the scratching time was increased stepwise by 2.5 min. (b, c) Scratching depth plotted as a function of normal load and scratching distance extracted from the wear patterns in a). The overall wear resistance of sandfish scale (*S. scincus*) against a sharp silicon tip is not superior to technical surfaces or to *S. diadema*.

The topography images at the top in Figure 7a present the wear patterns obtained in this way (load increased from left to right and top to bottom). The deflection sensitivity (*S*_ver_) varied for every pristine cantilever used for each sample. This effect causes a slight difference on normal load on each sample because we had to increase the loading force in voltage steps (*F*_load_ = *c_z_*·*S*_ver_·(*U*_setpoint_ − *U*_dis_)).

The topography images at the bottom of Figure 7a show the same type of experiment but in this case we kept the load constant (19.6 µN) but increased the scratching time in every scratched area. Abrasion resistance of samples can be evaluated by comparing the scratching depth revealed from the wear patterns topography images obtained after the scratching experiments.

Figure 7b summarizes the scratching depth as a function of normal load. All samples get finally scratched when normal load reaches a certain threshold but this value is different for every material. After reaching this threshold, the scratching depth increases nearly linearly with normal load. Figure 7c condenses the scratching depth versus scratching distance. The scratching depth increases constantly for the sandfish scale, Teflon, and PMMA. For aluminium the chosen threshold (19.6 µN) was too small to obtain any wear, so the scratching depth remained nearly zero. On the scale of the snake *S. diadema*, we find that scratching depth increases almost linearly at first but finally reaches a plateau. The same result occurred in the scratching experiment with fixed normal load (Figure 7b). We speculate that this effect might be caused by an inhomogeneity in the layer composition in the snake scale, i.e., a layer with higher wear resistance might be finally reached.

Comparing the results of the scratching experiments we conclude again that wear resistance of sandfish scales is not superior to other materials under investigation. At least on the microscale the tribological properties of sandfish scales do not reveal improved features.

### Microscale friction properties

**Figure 8:**
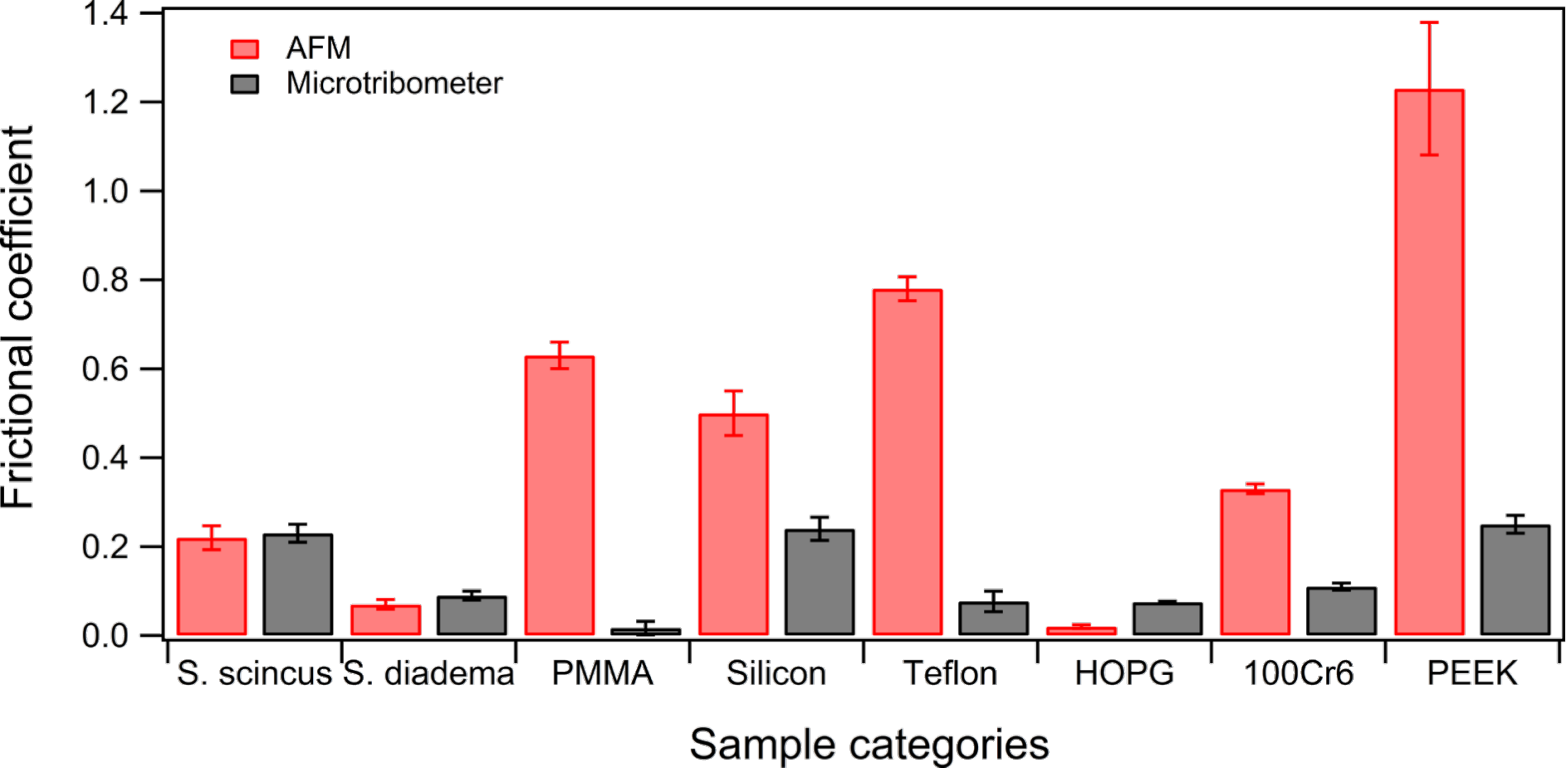
Comparison of the friction coefficients as measured by AFM and microtribometry in a sphere-on-plate reciprocating configuration. The diameter of the sapphire sphere was 1 mm. The materials tested were sandfish (*S. scincus*) and snake (*S. diadema*) scales as well as technical surfaces. For the latter PMMA, silicon, Teflon, HOPG, 100Cr6 bearing steel and PEEK were chosen as representatives.

In order to probe whether the results presented above and obtained by atomic force microscopy are a result of the inherent nanoscale nature of these experiments, or if they can be generalized to larger-contact scenarios, additional friction measurements with a reciprocating ball-on-plate microtribometer were conducted. Sandfish and snake scales together with technical surfaces such as Teflon, PMMA, HOPG, silicon, PEEK and 100Cr6 (AISI 5210) bearing steel were investigated. The results of these experiments are presented in Figure 8, plotted as the average friction coefficient for each of these surfaces, comparing the AFM and microtribometer results.

The comparison of friction coefficients between nanoscale and microscale experiments presented in Figure 8 demonstrates that also when in contact with a 1 mm sapphire sphere, biological surfaces, especially the sandfish scales, do not show superior frictional properties compared to technical surfaces. The example of the most common steel used in technical bearings (100Cr6, AISI 5210) has roughly the same friction coefficient as *S. diadema*. The friction coefficient of the sandfish scales is slightly higher. PEEK is a polymer widely used in tribological applications and it has approximately the same friction coefficient as the sandfish scales. Interestingly, some of the frictional coefficients obtained by atomic force microscopy are considerably higher as the ones recorded by microtribometry. All technical surfaces, with the exception of HOPG, exhibited considerably larger friction coefficients than the biological samples. These differences between tribological tests, conducted by AFM and microtribometry on technical surfaces, might be caused by submicron topography features present on these surfaces. Such components with a high wave vector of the power spectral density of a surface topography are most likely to have more influence on the nanoscale compared to the microscale [25].

## Conclusion

We analysed the tribological properties including adhesion, friction and resistance to abrasion of sandfish scales in detail utilizing various AFM techniques and probes with different size and surface chemistry. The experimental results do not indicate superior tribological properties of sandfish scales if compared to scales of other reptiles or technical surfaces. In agreement with classical theory the adhesion forces depend mainly on the tip size (or diameter) but adhesion on sandfish scales is not extraordinary small. The frictional coefficient measured with a sharp tip on a sandfish scale is interestingly lower than that on technical materials such as Teflon, PMMA or silicon but still larger than that on a common dry lubricant such as HOPG or a psammophile snake like *S. diadema*. Utilizing a spherical or sand probe results in the same overall outcome.

Abrasion resistance was characterised with two types of scratching experiments on scales of sandfish and the snake *S. diadema* in addition to three technical materials. Sandfish scales resist normal load better than most technical materials. However, they do not perform better over longer scratching periods. Consequently, tribology properties including adhesion, friction and abrasion resistance of sandfish scales are equal to the other samples investigated. In other words, it seems that sandfish scales do not feature outstanding tribological properties from the microscopic point of view. We, therefore, conclude that its scales are not the exclusive magic trick of the sandfish enabling its fabulous sand swimming.

Reviewing literature and our recent results it now seems likely that the dynamics of the sandfish locomotion as well as the elastic properties of the epidermis are important factors and not exceptional low friction and wear of the scales alone. Consequently, it will be important to consider not only scales but also the tissue underneath the epidermis as well as the dynamics of the swimming sandfish. Such experiments might hold the key for understanding the fabulous swimming abilities combined with low wear rates. It is possible that sandfish scales are not primarily designed to lower friction (a friction coefficient of 0.2 is comparably low for a dry sliding contact already) but to reduce wear in combination with the specific dynamics of sandfish. The latter is a significant technological challenge with high industrial impact that might lead to new robots which could swim through granular materials.
